# Advances in Nanoparticulate Drug Delivery Approaches for Sublingual and Buccal Administration

**DOI:** 10.3389/fphar.2019.01328

**Published:** 2019-11-05

**Authors:** Susan Hua

**Affiliations:** ^1^Therapeutic Targeting Research Group, School of Biomedical Sciences and Pharmacy, University of Newcastle, Callaghan, NSW, Australia; ^2^Hunter Medical Research Institute, New Lambton Heights, NSW, Australia

**Keywords:** buccal, sublingual, drug delivery, mucosal, formulation, nanoparticles, physiological factors, translation

## Abstract

The sublingual and buccal routes of administration have significant advantages for both local and systemic drug delivery. They have shown to be an effective alternative to the traditional oral route, especially when fast onset of action is required. Drugs can be rapidly and directly absorbed into the systemic circulation *via* venous drainage to the superior vena cava. Therefore, they are useful for drugs that undergo high hepatic clearance or degradation in the gastrointestinal tract, and for patients that have swallowing difficulties. Drugs administered *via* the sublingual and buccal routes are traditionally formulated as solid dosage forms (e.g., tablets, wafers, films, and patches), liquid dosage forms (e.g., sprays and drops), and semi-solid dosage forms (e.g., gels). Conventional dosage forms are commonly affected by physiological factors, which can reduce the contact of the formulation with the mucosa and lead to unpredictable drug absorption. There have been a number of advances in formulation development to improve the retention and absorption of drugs in the buccal and sublingual regions. This review will focus on the physiological aspects that influence buccal and sublingual drug delivery and the advances in nanoparticulate drug delivery approaches for sublingual and buccal administration. The clinical development pipeline with formulations approved and in clinical trials will also be addressed.

## Introduction

Drugs are generally administered in the oral cavity to either treat local conditions (e.g., infections and ulcers) or for the systemic absorption of drugs. In particular, the sublingual and buccal mucosal regions are highly vascularized and, therefore, are useful for systemic drug delivery. Sublingual administration involves placing a drug under the tongue and buccal administration involves placing a drug between the gums and cheek. The sublingual and buccal routes are considered promising alternatives to the traditional oral route for drug delivery.

**Figure 1:**
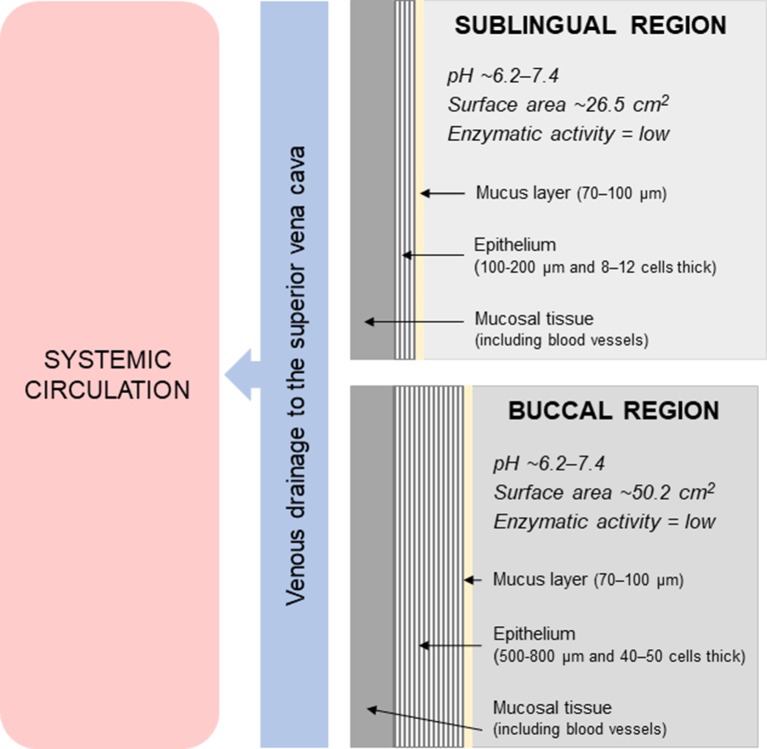
Schematic diagram of the sublingual and buccal regions in the oral cavity.

**Figure 1** shows a schematic diagram of the sublingual and buccal regions in the oral cavity. The oral cavity has a relatively neutral pH of approximately 6.2–7.4 and has limited enzymatic activity. The surface area of the oral mucosa is relatively small (100–200 cm^2^), with the sublingual and buccal regions having an estimated surface area of 26.5 ± 4.2 cm^2^ and 50.2 ± 2.9 cm^2^, respectively (Czerkinsky and Holmgren, 2012; Kraan et al., 2014). These regions in the oral cavity are lined by non-keratinized, stratified squamous epithelium that is 100–200 µm and 8–12 cells thick in the sublingual region, and 500–800 µm and 40–50 cells thick in the buccal region (Czerkinsky and Holmgren, 2012; Kraan et al., 2014). Components from the saliva also binds to the surface of the buccal and sublingual epithelium to create a mucus layer with an average thickness of 70–100 µm (Teubl et al., 2013). Underneath the epithelium is the lamina propria and submucosa that consists of connective tissue with a network of blood vessels, lymphatic vessels and smooth muscles. Drugs can be rapidly and directly absorbed into the systemic circulation *via* venous drainage to the superior vena cava.

A number of advances in drug formulation have been made in the area of sublingual and buccal drug delivery. This review will focus on the physiological aspects that influence buccal and sublingual drug delivery and the advances in nanoparticulate drug delivery approaches for sublingual and buccal administration. The clinical development pipeline with formulations approved and in clinical trials will also be addressed.

## Advantages and Disadvantages of the Sublingual and Buccal Routes for Drug Delivery

The sublingual and buccal routes of administration have a number of advantages (De Boer et al., 1984; Allen et al., 2011; Teubl et al., 2013), especially for systemic drug delivery. In general, they produce faster onset of action compared to orally ingested drug formulations. Drug absorption is relatively faster across the sublingual mucosa compared to the buccal mucosa due to the thinner epithelium. In addition to rapid absorption, the portion of drug that is absorbed through the blood vessels directly enters the systemic circulation and bypasses hepatic first-pass metabolic processes. Therefore, this route is particularly useful for highly soluble drugs that undergo high hepatic clearance or decomposition in the gastrointestinal tract. The non-adherent saliva in the buccal and sublingual regions also contains less mucin and limited enzymes (e.g., salivary amylase). Drugs may also be more stable owing to the pH in the mouth being relatively neutral compared to other parts of the gastrointestinal tract. Patients can easily self-administer doses and in most cases the effect of the drug can be quickly terminated, for example, by spitting out or swallowing the tablet. It is also beneficial for patients who suffer from swallowing difficulties.

In terms of disadvantages (De Boer et al., 1984; Allen et al., 2011; Teubl et al., 2013), the sublingual and buccal routes can be inconvenient for patients as it can involve some technical procedures to maintain the drug in the sublingual or buccal area for absorption without swallowing the drug. Not all drugs can be delivered *via* this route and generally only small doses can be administered. Drugs may also be unpalatable, bitter, or cause irritation to the oral mucosa, which may lead to voluntary expulsion or swallowing. Although the risk is low, there is a chance of accidental aspiration of the medication. Therefore, patients are recommended to be in an upright position when administering a dose. For similar reasons, sublingual or buccal medication should be avoided when a patient is unconscious or uncooperative. Furthermore, the buccal and sublingual routes are generally not suited or preferred for sustained drug release or for prolonged administration due to discomfort or inconvenience, especially when eating or drinking.

## Physiological Factors Influencing Sublingual and Buccal Drug Delivery

For effective drug delivery *via* the sublingual or buccal route of administration, several physiological factors should be considered in drug formulation design and development. These factors may influence drug bioavailability, stability, efficacy, and safety.

- *Residence time of the formulation:* Absorption is highly dependent on the residence time of the drug in the sublingual and buccal area. This may vary considerably depending on the formulation and the patient. Sublingual and buccal drugs are generally formulated as tablets, films, wafers, or sprays. The formulations differ in terms of need for disintegration and dissolution prior to drug absorption. In addition, patients should avoid eating, drinking, chewing, or swallowing until the medication has been absorbed (De Boer et al., 1984; Allen et al., 2011). Swallowing the medication will decrease the drug’s effectiveness. This can be particularly difficult for some patients, such as younger children.
- *Drug absorption:* For effective absorption to occur, the drug needs to have a balance between hydrophilic and lipophilic properties (De Boer et al., 1984; Allen et al., 2011; Brunton et al., 2018). That is, the drug needs to be soluble in aqueous buccal fluids and should also have high lipid solubility to be able to cross the epithelial membrane in these regions, which is usually by passive diffusion. This route is also more suitable for low to medium molecular weight drugs (De Boer et al., 1984; Allen et al., 2011; Brunton et al., 2018)— refer to examples in **Table 1**. In addition, drug absorption can be affected if the gums or mucosal membranes have open sores or areas of inflammation. This may lead to enhanced or irregular drug absorption and, therefore, should be avoided or used with caution. Conversely, smoking can decrease the sublingual or buccal absorption of medications due to vasoconstriction of the blood vessels.
- *pH of the saliva:* The pH of the saliva can affect drug absorption by affecting the ionization state of drugs. Drug molecules predominantly undergo passive absorption pathways *via* transcellular diffusion (through the cell) or paracellular diffusion (between cells), depending on their physicochemical characteristics (De Boer et al., 1984; Allen et al., 2011; Brunton et al., 2018). Transcellular diffusion is the most common mechanism and is usually proportional to the lipid solubility of the drug. Therefore, absorption is favored when the drug molecule is in the non-ionized form, which is much more lipophilic than the ionized form. For sublingual and buccal administration, this means that drugs with a high pKa value are preferred due to the relatively neutral pH of the saliva. Conversely, the paracellular pathway is favored for more hydrophilic or ionized molecules. It should be noted that the pH of the saliva can be temporarily altered by environmental (e.g., foods and drinks) or personal factors [e.g., oral disease (Baliga et al., 2013)], which can affect the sublingual and buccal absorption of drugs.
- *Flow of saliva:* Saliva flow can influence buccal and sublingual drug delivery by altering the rate of disintegration of the formulation and dissolution of the drug. For example, if the mouth is dry, this can negatively affect drug absorption. Conversely, if saliva flow is considerable, this can lead to the drug being swallowed before absorption. Saliva flow can be affected by age, medications (e.g., anticholinergic drugs), and medical conditions (e.g., Sjögren’s syndrome, cheilosis, glossodynia, dehydration, dysphagia, and problems with mastication) (Dawes, 1987; von Bultzingslowen et al., 2007).

**Table 1:** Sublingual and buccal formulations marketed and in clinical trials

Drug	Dosage form	Indication	Status
Lorazepam	Tablet	Sedation	Marketed *(Ativan)*
Zolpidem	Tablet	Insomnia	Marketed *(Edluar)*
Melatonin	Tablet	Insomnia	Marketed *(Melatonin Sublingual)*
Allergen extract	Tablet	Allergic rhinitis	Marketed *(Grastek, Oralair, Odactra, Ragwitek)*
Polyvalent mechanical bacterial lysate (biological)	Tablet	Chronic obstructive pulmonary disease	Marketed *(Ismigen)*
Isosorbide dinitrate	Tablet	Angina	Marketed *(Isordil)*
Sufentanil	Tablet	Pain	Marketed *(Dsuvia, Zalviso)*
Glyceryl trinitrate (nitroglycerin)	Tablet, spray	Angina	Marketed *(Anginine*, *Lycinate, Nitrolingual Pump Spray)*
Fentanyl	Tablet, spray, film, lozenge	Pain	Marketed *(Abstral, Actiq, Subsys, Fentora, Onsolis)*
Buprenorphine	Tablet, film	Pain	Marketed *(Temgesic, Belbuca)*
Nicotine	Tablet, film, gum, lozenge, spray	Smoking cessation	Marketed *(Nicabate, Nicotinell, Nicorette, QuitX, Nicaway, Nicabate Oral Strips, Nicorette QuickMist)*
Vitamin B12	Tablet, spray, oral liquid	Vitamin deficiency	Marketed *(Sublingual Vitamin B12)*
Desmopressin	Tablet, wafer	Nocturia	Marketed *(Minirin Melt, Nocdurna)*
Buprenorphine + naloxone	Film	Opioid dependence	Marketed *(Suboxone)*
Asenapine	Wafer	Schizophrenia	Marketed *(Saphris)*
Midazolam	Oral liquid (prefilled oral syringes)	Epilepsy	Marketed *(Buccolam, Epistatus)*
Nystatin	Oral liquid	Oral candidiasis	Marketed *(Nilstat, Mycostatin)*
Miconazole	Gel	Oral candidiasis	Marketed *(Daktarin, Decozol)*
Triamcinolone	Paste	Oral ulceration	Marketed *(Kenalog in Orabase)*
Zolmitriptan	Tablet	Cluster headache	Phase IV
Misoprostol	Tablet	Induction of labor, blood loss in myomectomy, abortion	Phase III/IV
Y-2 (adaravone and borneol)	Tablet	Healthy	Phase I
Alprazolam	Tablet	Anxiety disorder, sedation for endoscopy	Phase I/II/III completed
Riluzole	Tablet	Social anxiety disorder, amyotrophic lateral sclerosis	Phase I/II/III
Lobeline	Tablet	Methamphetamine dependence, Attention deficit disorder	Phase I/II
Cyclobenzaprine	Tablet	PTSD, fibromyalgia	Phase III
Olanzapine	Tablet	Schizophrenia	Phase IV completed
Agomelatine	Tablet	Major depressive disorder	Phase III completed
ALKS 5461	Tablet	Major depressive disorder	Phase III completed
Sildenafil	Tablet, wafer	Erectile dysfunction	Phase III completed
Cannabidiol	Tablet, oral liquid	Diabetic neuropathies, chronic pain, anxiety, inflammatory bowel disease	Phase I/II
Allergen extract (mite, artemisia annua, apple, birch pollen, grass pollen, blatella germanica, milk, peanuts, ragweed)	Oral liquid	Atopic dermatitis, allergic rhinitis, allergic conjunctivitis, food hypersensitivity	Phase I/II/III/IV
Influenza vaccine	Oral liquid	Healthy	Phase I completed
Naloxone	Oral liquid	Chronic pruritus	Phase I/II completed
Ketorolac	Oral liquid	Postoperative pain	Phase IV
Oral enterotoxigenic Escherichia coli vaccine (biological)	Oral liquid	Gastroenteritis Escherichia coli	Phase I
Cholera toxin B subunit (biological)	Oral liquid	Healthy	Phase I completed
UISH001	Oral liquid	Urinary incontinence	Phase I/II completed
Methadone	Oral liquid	Cancer Pain	Phase I completed
Cyclobenzaprine	Oral liquid	Healthy	Phase I completed
Tacrolimus	Oral liquid, powder	Bone marrow transplant, organ transplant, chronic renal failure	Phase IV
Ticagrelor	Powder, tablet	Acute coronary syndrome, percutaneous coronary intervention	Phase IV
Tizanidine	Powder	Muscle spasticity	Phase I/II completed
Polyoxidonium	Spray	Acute respiratory infection	Phase III
Flumazenil	Spray	Healthy	Phase I/II completed
Artemether	Spray	Plasmodium falciparum malaria	Phase III completed
Insulin	Film, spray	Healthy, type 1 diabetes, Type 2 diabetes	Phase I/III
Ketamine	Film, wafer	Healthy, pain	Phase I/II completed
Dexmedetomidine	Film	Schizophrenia	Phase I
Apomorphine	Film	Parkinson’s disease	Phase II/III
Montelukast	Film	Alzheimer disease	Phase II
Diazepam	Film	Epilepsy	Phase III
NTG1523 (nitroglycerin)	Rapid absorbable capsule	Angina pectoris	Phase IV
Ropivacaine	Liposomal gel	Topical anesthesia	Phase I completed

(Ref: clinicaltrials.gov; ema.europa.eu; fda.gov; tga.gov.au; drugs.com).

## Nanoparticulate Drug Delivery Approaches

Nanoparticulate systems have previously been shown to improve the accumulation, uptake, and absorption of drugs across a variety of biological barriers, including the skin (Hua, 2015) and gastrointestinal tract (Hua et al., 2015). Therefore, it was inevitable for nanoparticles to be investigated for sublingual and buccally drug delivery. Nanoparticulate dosage forms differ from conventional dosage forms by loading the drug or active compound into nanoparticles prior to dispersion in a formulation base. They have been incorporated into various dosage forms for sublingual and buccal drug delivery, including gels (Marques et al., 2017), sprays (Baltzley et al., 2018), tablets (Gavin et al., 2015; El-Nahas et al., 2017), films (Giovino et al., 2013; Mortazavian et al., 2014; Al-Dhubiab et al., 2016; Masek et al., 2017; Castro et al., 2018a; Mahdizadeh Barzoki et al., 2018; Al-Nemrawi et al., 2019), and patches (Mahdizadeh Barzoki et al., 2016). These nanoparticulate formulations have been shown to: (i) improve drug permeability across the epithelium; (ii) modify drug release kinetics (e.g., controlled release or sustained release); (iii) provide solubilization (i.e., to deliver compounds which have physicochemical properties that strongly limit their aqueous solubility); and/or (iv) protect compounds that are sensitive to degradation (e.g., peptides) (Morales and Brayden, 2017; Hua et al., 2018). These factors all aim to promote higher sublingual or buccal bioavailability of drugs for subsequent systemic absorption.

For nanoparticulate dosage forms to be effective for sublingual or buccal drug delivery, two main factors should be considered. Firstly, the physicochemical properties of the nanoparticles themselves (e.g., size, charge, composition, and surface properties) for optimal interaction with the sublingual or buccal mucosa. A number of different nanoparticulate systems have been evaluated for sublingual and buccal drug delivery, with polymer-based and lipid-based compositions being the most common (He et al., 2009; Roblegg et al., 2012; Teubl et al., 2013; Teubl et al., 2015; Mouftah et al., 2016; Patil and Devarajan, 2016; Chaves et al., 2017; Xu et al., 2018). The composition and structure of nanoparticles can be designed to confer a number of different properties, including mucoadhesion, bioadhesion, mucus-penetration, controlled release, and deformability (Hua et al., 2015). For example, inclusion of a hydrophilic polyethylene glycol (PEG) coating to the surface of nanoparticles has been shown to reduce its interaction with the mucus constituents, increase particle translocation through the mucus and mucosa, and enhance its delivery into lymph nodes (Wang et al., 2008; Hua et al., 2015; Masek et al., 2017).

In terms of optimal nanoparticle size for sublingual or buccal administration, most of the studies in this area have used nanoparticles between approximately 100 to 300 nm in size. Very few studies have comprehensively evaluated a range of particle sizes for optimal interaction with the buccal or sublingual mucosa. For example, Teubl et al. (2013) demonstrated in *ex vivo* studies using porcine buccal mucosa that neutral polystyrene nanoparticles (25, 50, and 200 nm) dispersed in an aqueous base were able to penetrate into the mucosal tissue intact, with the 200-nm sized nanoparticles penetrating more rapidly and into deeper regions of the mucosa. It was suggested that the smaller nanoparticles were readily entrapped and immobilized in the mucus network. This is also supported by Holpuch et al. (Holpuch et al., 2010) which showed that 200-nm nanoparticles (FluoSpheres^®^ polystyrene nanoparticles) were able to penetrate through the epithelium and basement membrane into the underlying connective tissue of intact normal human oral mucosal tissues that were obtained from patients undergoing surgical procedures. It should be noted that both studies used polystyrene nanoparticles, which are unable to be metabolized and can interfere with cell metabolism pathways (Holpuch et al., 2010). Therefore, further studies would be useful to evaluate the effect of more clinically translatable nanoparticulate compositions over a range of particle sizes for mucosal permeability and drug absorption for sublingual and buccal drug delivery.

There are conflicting results regarding the influence of surface charge on nanoparticle interaction with the oral mucosa. Roblegg et al. (2012) showed that 20 nm anionic (negatively charged) and 200 nm cationic (positively charged) nanoparticles were both able to permeate the mucus layer of porcine buccal mucosa. The cationic nanoparticles (200 nm) penetrated deeper into the buccal mucosal tissue compared to the 20 nm anionic nanoparticles, which remained in the top third region of the epithelium. The study reported that 200 nm anionic nanoparticles were entrapped within the mucus, formed agglomerates, and were unable to penetrate the epithelium. Similar differences in the interaction of the mucosa with nanoparticles of opposite charges were observed by Chaves et al. (2017). However, other studies have reported that cationic nanoparticles interacted more with the mucus and exhibited lower mucosal permeability in comparison to anionic nanoparticles (Chen et al., 2010; Yuan et al., 2011; Mouftah et al., 2016; Patil and Devarajan, 2016; Xu et al., 2018). This is also supported by studies in the lower gastrointestinal tract, whereby electrostatic interaction between cationic nanoparticles and the negatively charged mucins impeded the transport of the nanoparticles through the mucus layer (Hua et al., 2015). Anionic nanoparticles were able to interdiffuse among the mucus network due to less electrostatic interaction with the mucus (Hua et al., 2015).

The second main factor that should be considered for effective sublingual or buccal drug delivery is the interaction of the nanoparticles with the formulation base. The nanoparticles should be stable when incorporated into the pharmaceutical base, especially during manufacturing and storage. In addition, the formulation base should increase the residence time of the formulation in the sublingual or buccal region to optimize drug permeability and systemic absorption. There are inconsistent results as to the actual interaction of the nanoparticle-embedded formulations with the mucosal tissue. The majority of the studies have demonstrated sustained drug release from the nanoparticles embedded in the dosage form, with the drug then being diffused into the formulation base and absorbed into the adhered mucosa. These include nanoparticles incorporated into gels (Marques et al., 2017), sprays (Baltzley et al., 2018), tablets (Gavin et al., 2015; El-Nahas et al., 2017), films (Giovino et al., 2013; Mazzarino et al., 2014; Mortazavian et al., 2014; Al-Dhubiab et al., 2016; Masek et al., 2017; Castro et al., 2018a; Mahdizadeh Barzoki et al., 2018; Al-Nemrawi et al., 2019), and patches (Mahdizadeh Barzoki et al., 2016). Very few studies have demonstrated release of nanoparticles from the formulation base and mucosal penetration of intact nanoparticles for drug delivery (Mortazavian et al., 2014; Masek et al., 2017). For example, Masek et al. (2017) developed nanofiber-based mucoadhesive films consisting of an electrospun nanofibrous reservoir layer (with nanoparticles reversibly adsorbed to the surface of the nanofibers or deposited in the pores between the nanofibers), a mucoadhesive film layer, and a protective backing layer. The results from both *ex vivo* and *in vivo* studies in pigs demonstrated that the nanofibrous mucoadhesive films were able to avoid rapid clearance of nanoparticles from the site of application, maintain a long-term concentration gradient of nanoparticles at the mucosal surface, and ensure unidirectional diffusion of nanoparticles towards mucosal surfaces. Histological samples excised 2 h after *in vivo* administration showed penetration of intact nanoparticles into the mucosa as well as regional lymph nodes.

The reasons for the discrepancy in the mechanism of action of nanoparticles when administered in a liquid base (e.g., water or buffered solution) or embedded into a formulation base (e.g., films, gels, and tablets) for sublingual or buccal drug delivery are still incompletely understood. Further studies are needed to determine whether it is more beneficial for nanoparticles to be used as a scaffold to promote stability and control drug release kinetics from within the formulation base or following mucosal penetration as intact particles. The former mechanism would place more importance on the retention of the formulation base to the mucosa and the stability of the nanoparticles in the formulation base for drug release, whereas the latter mechanism would place more importance on the physicochemical characteristics of the nanoparticles themselves for mucosal penetration. Most of the studies have only been conducted in *in vitro* and/or *ex vivo* models, with very limited *in vivo* studies available. *In vivo* studies provide better insights into the real-time performance of the formulation, as drug absorption is affected by a number of physiological factors as discussed earlier. In addition, there are significant anatomical differences in the sublingual and buccal mucosa among species. Porcine mucosa is the most similar to human mucosa and is widely used in *ex vivo* studies, however it is more common to use rodents in *in vivo* studies which have keratinized mucosa (Masek et al., 2017). Keratinization of the mucosa acts as an additional barrier for the penetration of drugs and nanoparticles, which should be taken into account when evaluating the results. Although the results to date support the use of nanoparticulate drug delivery approaches for sublingual and buccal administration, further comprehensive mechanistic and preclinical studies are required to ensure reproducibility of efficacy and safety outcomes.

## Sublingual and Buccal Formulations Approved and in Clinical Trials

A number of sublingual and buccal formulations are on the market with more in clinical development. **Table 1** shows examples of the sublingual and buccal formulations that are approved or in clinical trials. Those approved for clinical use have varied indications that also benefit from faster onset of action, including sedation, insomnia, angina, pain, and smoking cessation. The drugs incorporated vary in their therapeutic index as well as their duration of use, which indicate the prospect of using drugs with a narrow therapeutic index and for long-term therapy. Biologics have also made its way into the market with the delivery of allergen extracts and polyvalent mechanical bacterial lysate for use in allergic rhinitis and chronic obstructive pulmonary disease (COPD), respectively. Sublingual and buccal formulations approved for clinical use generally incorporate drugs in conventional dosage forms such as solid dosage forms (e.g., tablets, wafers, lozenges, and films), liquid dosage forms (e.g., sprays and oral liquid drops), and semi-solid dosage forms (e.g., gels and paste) (Allen et al., 2011). Solid dosage forms are typically manufactured to disintegrate or dissolve rapidly in a small quantity of saliva to allow fast drug absorption through the mucosa, without the need for water. In contrast, liquid dosage forms for sublingual and buccal use contain the drug dissolved (solution) or dispersed (suspension) in a vehicle. This is then administered as oral liquid drops or sprays, with the latter typically having a metered valve to control the dose of the drug delivered.

The majority of the formulations in clinical trials (**Table 1**) incorporate already approved drugs or novel compounds into conventional sublingual and buccal dosage forms—in particular, tablets, films, and oral liquids. It should be noted that drugs evaluated in the early phases of clinical investigation are commonly administered as a powder or oral liquid. Powders are typically formulated by opening clinically available capsules or crushing tablets, whereas oral liquids are attained by dispersing the powder into a liquid base or using the parenteral formulations of the drug. These studies are mainly focused on evaluating the pharmacokinetics and efficacy of the drug following sublingual or buccal administration, rather than assessing the performance of novel formulations.

Very few innovative dosage forms for sublingual and buccal drug delivery have reached the clinical development phase. The main strategies have been the incorporation of permeation enhancers or mucoadhesive constituents to conventional dosage forms. Conventional dosage forms are commonly affected by physiological factors (e.g., saliva and swallowing), which can reduce the contact of the formulation with the mucosa and lead to unpredictable drug absorption. In addition, the multicellular thickness and stratified nature of the sublingual and buccal epithelium can contribute to reduced drug absorption across these regions. These strategies have been shown to improve mucosal retention and/or permeability of conventional dosage forms. For example, permeation enhancers (e.g., surfactants, bile salts, fatty acids, cyclodextrins, and chelators) have been shown to improve the mucosal permeability and absorption of various compounds (Tsutsumi et al., 1998; Shojaei et al., 1999; Bird et al., 2001; Burgalassi et al., 2006; Sohi et al., 2010; Tian et al., 2012; Prasanth et al., 2014; Patil and Devarajan, 2014; Ojewole et al., 2014; Marxen et al., 2018) by: (i) changing mucus rheology; (ii) increasing the fluidity of the lipid bilayer membrane; (iii) acting on the components at tight junctions; (iv) inhibiting mucosal enzymes; and (v) increasing the thermodynamic activity of drugs (Chinna Reddy and Chaitanya, 2011). In addition, the incorporation of mucoadhesive constituents has been demonstrated to enhance formulation retention time with the sublingual or buccal mucosa (Das and Das, 2004; Razafindratsita et al., 2007; Perioli and Pagano, 2013; Ikram et al., 2015; Yildiz Pekoz et al., 2016; El-Nabarawi et al., 2016; Ammar et al., 2017; Parodi et al., 2017; Celik, 2017; Salehi and Boddohi, 2017; Vasseur et al., 2017; Khan and Boateng, 2018; Razzaq et al., 2018; Sharma et al., 2018). This has been done primarily for solid dosage forms and semi-solid dosage forms. In particular, mucoadhesive polymers are commonly used in these formulations, including synthetic polymers (e.g., cellulose derivatives and poly(acrylic acid)-based polymers) and those from natural sources (e.g., chitosan, hyaluronic acid, agarose, and various gums). An impermeable backing layer may be incorporated in solid dosage forms (e.g., films, patches, and tablets) to allow unidirectional drug delivery (Guo and Cooklock, 1996; Benes et al., 1997; Shojaei et al., 1998; El-Nabarawi et al., 2016).

It is expected that more innovative dosage forms will eventually reach clinical trials following comprehensive preclinical assessment and optimization. This includes nanoparticulate formulations, especially for the systemic delivery of drugs. Ropivacaine liposomal gel is the only nanoparticulate formulation that has reached clinical studies for sublingual and buccal drug delivery. It has been evaluated for local drug delivery as a topical anesthetic in Phase I clinical studies. Furthermore, slow-disintegrating and non-disintegrating dosage forms, particularly for buccal drug delivery, have been extensively investigated in the literature to extend or control the release of active substances over a prolonged period (Scholz et al., 2008; Bahri-Najafi et al., 2014; Kaur et al., 2014; Jaipal et al., 2016; Celik, 2017; Lindert and Breitkreutz, 2017; Celik et al., 2017; Farag et al., 2018; Castro et al., 2018b). For example, multilayered films have been developed for controlled drug delivery and are generally designed to remain in their form and slowly release drug over a specified time (Lindert and Breitkreutz, 2017). It should be noted that formulations that have prolonged contact with the mucosa may cause irritation and/or discomfort for the patient, especially with concurrent eating or drinking. There is also a possibility for the dosage form to detach from the mucosa and be swallowed, which can lead to subsequent adherence to other parts of the gastrointestinal tract (e.g., esophagus). The results from clinical studies will determine the feasible of these dosage forms in clinical practice.

## Conclusion

The sublingual and buccal routes of administration have significant advantages for systemic drug delivery. They have shown to be an effective alternative to the traditional oral route, especially when fast onset of action is required. In addition, they are useful for drugs that undergo high hepatic clearance or degradation in the gastrointestinal tract, and for patients that have swallowing difficulties. Although significant advances in drug formulation have been reported in the literature, particularly to improve retention and absorption in the buccal and sublingual regions, very few of them have translated to the clinical phase. For clinical translation to be justified, there needs to be a clear benefit of efficacy and/or safety with any new drug formulation compared to clinically available dosage forms (Hua et al., 2018). In addition, comprehensive evaluations of the pharmacokinetics, stability, efficacy, and safety of the formulations are required in appropriate animal models as well as in clinical studies, based on regulatory standards and protocols. For innovative platforms, such as nanoparticles, mechanism of action and safety of the different carriers following mucosal interaction and/or uptake need to be explored further (Bergin and Witzmann, 2013; Talkar et al., 2018; Vita et al., 2019). Complexity in drug formulation is also a key factor that can be a barrier to clinical translation, irrespective of its therapeutic efficacy (Hua et al., 2018). Therefore, simplification in formulation design is required to allow efficient and reproducible large-scale manufacturing. The availability of standardized testing methods can also be a limitation to reliably assess the quality of more complex or innovative formulations for regulatory standards.

## Author Contributions

SH was involved in conception of the idea for the review, drafted the manuscript, and approved the final version of the manuscript.

## Conflict of Interest

The author declares that the research was conducted in the absence of any commercial or financial relationships that could be construed as a potential conflict of interest.
